# Supplementary material for: Failure of Micractinium simplicissimum Phosphate Resilience upon Abrupt Re-Feeding of Its Phosphorus-Starved Cultures
Source: Int J Mol Sci. 2023 May 9;24(10):8484. doi: 10.3390/ijms24108484 (PMC10218051; doi:10.3390/ijms24108484)
Supplement: Supplementary file 1 [file ijms-24-08484-s001.zip › ijms-2383337-supplementary.pdf]

**Table S1.** The JIP-test parameters used for monitoring of the microalgal cultures in the present work (according to [54,55]).

| Parameter | Description |
|---|---|
| $F_O$, $F_J$, $F_{300}$, $F_m$ | Fluorescence yield at the points O (minimal fluorescence, when all PS II reaction centers, RC are open), J (2 ms), at 300 μs (K-step), and at the point of the maximum of OJIP, M, when all PS II RC are closed |
| PS II $Q_y = \varphi_{P_0} = \frac{F_m - F_O}{F_m} = \frac{F_V}{F_m}$ | Maximum potential quantum yield of primary photochemistry in photosystem II |
| $\mathrm{NPQ} = \frac{F_m}{F_m'} - 1$ | A non-photochemical quenching parameter according to Stern-Volmer |
| $\psi_0 = 1 - \frac{F_J - F_O}{F_M - F_O}$ | The probability of electron transfer from PS II to PQ pool |
| $M_0 = 4\frac{F_{300} - F_O}{F_M - F_O}$ | An approximation of the initial slope of OJIP attributed to QA reduction |
| $\phi_{D_0} = \frac{F_O}{F_M}$ | Quantum yield of energy dissipation |
| $\phi_{E_0} = \psi_0\left(1 - \frac{F_O}{F_M}\right)$ | Quantum yield of electron transport |
| $\mathrm{ABS/RC} = M_0 \cdot \frac{1}{V_J} \cdot \frac{1}{\varphi_{P_0}}$ | Absorbed energy flux per RC |
| $\mathrm{TR_0/RC} = M_0 \cdot \frac{1}{V_J}$ | Trapped energy flux per RC |
| $\mathrm{DI_0/RC} = \mathrm{ABS/RC} - \mathrm{TR_0/RC}$ | Thermally dissipated energy flux per RC |
| $\mathrm{PI_{ABS}} = \mathrm{ABS/RC} \cdot \left(\frac{\varphi_{P_0}}{1-\varphi_{P_0}}\right) \cdot \left(\frac{\psi_0}{1-\psi_0}\right)$ | Performance index expressed on the light absorption basis |

**Table S2**. Elemental composition and localization the inclusions in the ruptured cells *Micractinium simplicissimum* IPPAS C-2056 (for explanation, see text)

| Medium type or time after $P_i$ re-feeding | Cell wall | Cytosol | Vacuole | | Chloroplast stroma | Nucleus |
|---|---|---|---|---|---|---|
| | | | Small spherules | Large globules | | |
| BG-$11_K$ | ND | ND | ND | ND | ND | ND |
| P-free medium | ND | ND | ND | ND | ND | ND |
| 4 h* | ND | ND | ND | ND | ND | ND |
| 24 h | **Fe,<br>Fe-P,<br>Fe-P-S,<br>P-Ca-Mg,<br>P-Ca,<br>P-Mg,<br>P† | P-Na-Mg-Ca,<br>P-N-Mg | P-Mg-Ca†<br>P-N-S-Ca-Mg<br>P-N-Mg | P-N-Mg,<br>P-N-Ca | P-S†,<br>P-Mg-Ca | P† |
| 72 h | Fe,<br>P,<br>S,<br>P-Fe-Na-Ca | ND | ND | ND | ND | ND |
| 168–240 h | P | P-N-Mg,<br>P-N,<br>P | P-Na-Mg,<br>P-Na-Mg-Ca | ND | P-N | ND |

*Time after re-feeding of the P-starved cultures with $P_i$.

**Elements are ordered according to an increase in the magnitude of the corresponding element peak in the EDX spectrum.

†The EDX spectrum possessed the peak of uranium (U).

ND—no data.
